# Comparison of diet consumption, body composition and lipoprotein lipid values of Kuwaiti fencing players with international norms

**DOI:** 10.1186/1550-2783-8-13

**Published:** 2011-10-12

**Authors:** Kazem Ghloum, Salman Hajji

**Affiliations:** 1 Department of Physical Education and Sport, College of Basic education, The Public Authority for Applied Education and Training, Aladailia-Block 4-3rd ring Road., The State of Kuwait

## Abstract

**Background:**

No published data is currently available that describes the dietary patterns or physiological profiles of athletes participating on the Kuwaiti national fencing team and its potential impact on health and physical performance. The purpose of this investigation was to: 1) collect baseline data on nutrient intake 2) collect, analyze and report baseline for body composition, plasma lipid and lipoprotein concentrations during the competitive season, 3) compare the results with the international norms, 4) and provide necessary health and nutritional information in order to enhance the athletes' performance and skills.

**Methods:**

Fifteen national-class fencers 21.5 ± 2.6 years of age participated in this study. Food intake was measured using a 3-day food record. Body composition was estimated using both the BOD POD and Body Mass Index (BMI). Total blood lipid profiles and maximum oxygen consumption was measured for each of the subjects during the competitive season.

**Results:**

The results of the present study showed significant differences in dietary consumption in comparison with the recommended dietary allowances (RDA). The blood lipids profile and body composition (BMI and % body fat) were in normal range in comparison with international norms However, the average VO_2 max _value was less than the value of the other fencers.

**Conclusion:**

Due to the results of the research study, a dietary regimen can be designed that would better enhance athletic performance and minimize any health risks associated with nutrition. Percent body fat and BMI will also be categorized for all players. In addition, the plasma blood tests will help to determine if any of the players have an excessive level of lipids or any blood abnormalities. The outcomes of present study will have a direct impact on the players health and therefore their skills and athletic performance.

## Background

Fencing is an open-skilled combat sport that was admitted to the first modern Olympic games in Athens 1896. Modern fencing competition consists of three different weapons: the foil, the sabre and the epée, each contested with different rules. The actual matches represent only 18% of total competition time, with effective action time being 17 and 48 minutes. The physical demands of competitive fencing require a high level of aerobic and anaerobic conditioning.

It is well recognized that athletic performance is enhanced by optimal nutrition (American College of Sports Medicine, American Dietetic Association, and Dietitians of Canada, 2009) [1]. Research has demonstrated that athletes are interested in nutritional information, while sport nutrition information is becoming more available [2-6].

There a strong positive correlation between food intake, body composition and blood lipid levels. Nevertheless, nutrition-related knowledge deficits and dietary inadequacies persist among many Kuwaiti athletes [7-9]. Fencing athletes remain uneducated about proper nutrient supplementation and dietary habits. Many diets include high intake of processed and refined foods along with great amount of saturated fats and very low intake of fresh fruits and vegetables. Such diets contribute to overall poor health and impair athletic performance [10-12].

Body composition, is an important aspect in relation to an athlete's performance [10]. The ideal body composition varies by sport, but in general, the less fat mass, the greater the performance potential. Previous studies [13,14] have demonstrated that success in fencing depends more on technique, speed, and agility as opposed to a high aerobic capacity and low percent body fat percentage. Although the findings of the study may be true, numerous studies [15-17] confirmed that aerobic training increases the fencers' reaction times, their attention capacities and causes an overall lower body fat composition. Furthermore, body fat distribution has been associated with atherosclerotic disease risk factors as well as injuries associated with back, knees, ankles joints and muscles problems [18-20].

Measurements of body composition are valuable tools when determining appropriate nutritional intakes, since there is a direct relationship between dietary intake and body composition [21-23]. Excessive levels of body fat can indicate an inadequate amount of time spent in general physical preparation especially aerobic training, or an unbalanced dietary intake.

Blood lipids test is a tool used by physicians to detect potentially harmful and evolving conditions, such as heart disease. There is strong agreement that physical activity lowers the risk of cardiovascular diseases (CVD) and that part of this risk reduction involves positive changes in plasma lipids and lipoproteins [14,16,24-29].

The significance of understanding body composition, dietary intake, and blood lipid values of these athletes may lead to improved health and physical performance as well as early identification of health abnormalities.

A review of current scientific literature revealed that no research papers have yet been published describing the dietary patterns and physiological profiles of the Kuwaiti national fencing team; therefore, the purpose of this investigation was to 1) collect baseline data on nutrient intake in order to advise athletes about nutrition practices that might enhance performance, 2) collect, analyze and report baseline data for body composition, plasma lipid and lipoprotein concentrations during the competitive season, 3) compare the results with international norms, and 4) make health and nutritional recommendations, in order to enhance fencing players physical performance and skills, and to reduce potential future health risks.

## Methods

### Subjects

Fifteen (n = 15) male national-class fencers aged 21.5 ± 2.6 years were selected for this investigation. These athletes were recruited from the Kuwait national fencing team. Each subject performed approximately 10-12 h of practice per week (at least 2 h of training per day and a competition match during the weekend). Prior to the study, the purpose and objective of this research were carefully explained to each subject and the coaching staff. Data was collected between January and March of 2010 during the competitive season.

### Body composition

Body composition was estimated by two methods in this investigation. Body mass index (BMI) was used to determine weight relative to height and obesity related health risks. Weight and height were measured to the nearest 0.1 kg and 0.1 cm, with a Seca portable height stadiometer (Leicester, England). BMI was calculated using the following formula: weight (kg)/[height (m)]^2^. Percentage body fat was estimated using the BOD POD air-displacement plethysmography (ADP) (Life Measurement, Inc, Concord, CA) device within 24 hours before the study began. The BOD POD is considered a reliable method of assessing body composition and has been validated through many independent research studies [30-34]. However, in some subjects, 2-3 measurements were needed to obtain a satisfactory result. The full test required 3-5 minutes to complete and body fat percentage was automatically calculated by the computer; body density was calculated as mass/body volume and body fat percentage was calculated by using Brozek's formula [35].

### Dietary analysis

A three-day dietary record was used to estimate mean daily dietary intake. Food models, household measuring utensils (e.g., teaspoon, tablespoon, and cup), sport drink containers, and packaged foods commonly consumed, were used by the researchers during each meeting to visually illustrate portion sizes. Dietary analysis was performed using a commercially available software program (DINE Systems, Inc software package; North Carolina, USA). All evaluations were analyzed by one researcher to ensure accuracy and consistency [36]. The analysis provided detailed information about the calories required, and intake of carbohydrates (complex, simple and fiber), lipids (saturated, monounsaturated, and polyunsaturated) and proteins. They were compared with the recommendations proposed by the American Dietetic Association (ADA), Dieticians of Canada (DC), and American College of Sports Medicine (ACSM)[1]. Dietary fiber, cholesterol, vitamin C, and the minerals: sodium, calcium, potassium, phosphorus and iron were compared with the values recommended by the dietary reference intake (DRI) [37]. The unit of analysis was the average of the sum of nutrient intake over three days. This program calculates the absolute measure of the quantity of each nutrient (in grams, milligrams, or micrograms) and the corresponding percentages to RDA. Each athlete's diet recommendations were considered in the present study.

To determine the caloric requirement for the Kuwaiti fencers, a basal metabolic rate (BMR) was calculated using Harris Benedict equation [38]. This formula considered the factors of height, weight, age, and sex as well as a physical activity level of 1.5 × BMR. As a result, the mean caloric intake for Kuwaiti fencers was 2655 calories/day. Subjects were asked to record their entire food intake carefully. Furthermore, at the time of this data collection, all subjects were apparently healthy and none were taking medications known to alter food and energy metabolism. Subjects were instructed not to modify their food intake or eating patterns throughout the study. The days recorded consisted of two days of training followed by a day of rest.

### Blood lipid profile

All subjects were reported to a commercial biomedical Laboratory (HBM Inc, Kuwait) after a 12 hour overnight fast. Blood samples were drawn from the antecubital vein. Serum total cholesterol and triglycerides were analyzed by enzymatic techniques in a Hitachi 911/904 (Roche Diagnostics, Basel, Switzerland) according to the manufacturer's protocol. The high density lipoprotein fraction of cholesterol (HDL-C) was measured after precipitation of the very low density lipoprotein (VLDLC) and low density lipoprotein (LDL-C) fractions with phosphotungstic acid. LDL-C was precipitated with Biomerieux reagent. Hemoglobin values were measured using an automatic multi-parameter blood cell counter (Sysmex^® ^KX-21).

### Maximal Oxygen Consumption (VO_2 max_)

*VO_2 max _*was assessed using a modified Bruce protocol. This protocol began after a 2-min warm-up. Treadmill speed, grade, or both were increased every 2 minutes until cardiopulmonary fatigue was reached and O_2 _max was obtained. Criteria for attainment of *VO_2 max _*included a < 2 ml/kg increase in oxygen consumption (O_2_) with an increased work rate, a respiratory exchange ratio (RER) greater than or equal to 1.1, and/or the subject's inability to maintain this work rate. *VO_2 max _*is expressed in ml/kg/min.

### Statistical analysis

All data were presented as mean, standard deviations (SD) and ± standard errors of the mean (SEM). Differences in mean values of the Kuwaiti fencers in body composition and blood lipids profile were analyzed using the average of the sum of the normal range and by applying a one sample t-test. In addition, the mean dietary intake of different foods and *V*O_2 max _values were compared using the one sample t-test. All the variables were compared with the international norm applying a t-test for independent samples. A probability value of ≤ 0.05 was considered significant. Data was analyzed using the Statistical Package of Social Sciences (SPSS) version 17 (Chicago, IL).

## Results

The results of the present study showed a statistically significant difference in dietary consumption between the athletes daily average nutrient intake and the recommended dietary allowances (RDA) The blood lipids profile, body composition (BMI and %body fat), and *V*O_2 max _were within the normal range in comparison with international norms.

A complete description of the fencing players physical characteristics (mean and standard deviation), including age, height, weight, body mass index, percent body fat, and maximum oxygen consumption are illustrated in Table 1.

**Table 1:** Baseline characteristics of Kuwaiti fencing players (means ± SD)

N	Players ID	Age (years)	Height (cm)	Weight (kg)	BMI (kg/m^2^)	% Body Fat	*V*O_2 max _(ml.kg^-1^.min^-1^)
1	MK	24.2	181.2	77.2	23.6	13.3	52.6

2	AN	21.2	179.2	75.3	23.5	14.5	43.3

3	SS	20.1	178.4	61.5	19.4	10.3	60.6

4	FM	19.5	181.0	78.1	23.8	15.8	54.4

5	AD	19.8.	177.3	65.6	19.8	8	46.8

6	AA	27.2	165.8	63.4	23.2	12.7	48.9

7	AM	18.9	178.6	56.5	17.8	6	54.6

8	AAS	18.4	181.2	58.5	17.1	6	49.9

9	AAK	25.1	174.3	64.5	21.3	15.3	51.6

10	AAF	24.6	165.2	72.1	26.5	17.7	47.9

11	MA	22.1	182.1	119.1	35.9	29.3	46.4

12	AJ	21.2	171.6	61.2	27.3	11.7	50.2

13	EA	19.2	167.3	69.1	24.7	16.3	43.2

14	AAB	20.1	178.3	77.0	24.3	18.2	47.8

15	KA	22.4	167.5	68.1	24.4	10.1	44.8

Χ		21.5	175.2	71.1	23.5*	13.9*	49.6

S.D.		2.6	6.1	15.0	4.54	5.95	4.76

S.E.		.68	1.58	3.84	1.17	1.59	1.23

*p *< 0.05 significant different between the present study and international norms

N = numbers of subjects

BMI = Body Mass Index, %Fat = percentage of body fat, *V*O_2 max _= maximum Oxygen Consumption (ml.kg^-1^.min^-1^)

**Table 2:** Blood profiles of all subjects (n = 15)

Variables	Fencing Players (mean ± SD)	Normal Range	Mean of Normal Range
Glucose (mmol/L)	4.91 ± .33	3.9-6.38	5.14

Triglycerides (mmol/L)	1.13 ± .53	0.40-2.50	1.45

Total Cholesterol (mmol/L)	3.87 ± .16	1.3 - 6.24	3.77

HDL-C (mmol/L)	1.06 ± .23	0.91 - 1.56	1.23

LDL-C (mmol/L)	2.32 ± .55	< 3.4	< 3.4

HGB (mmol/L)	15.13 ± .61	14.0 - 17.5	15.75

Values are mean ± SD.

Abbreviations: HDL = high density lipoprotein; LDL = low density lipoprotein; HGB = hemoglobin;

Normal range according to National Heart, lung and blood institute. U.S. Department of Health and Human Services.

The mean age of Kuwaiti male fencers was 21.5 ± 2.6 years with an average height and weight of 175.2 ± 6.1 and 71.1 ± 15.0 respectively.

The mean BMI and % body fat for Kuwaiti fencers was 23.5 ± 4.54 and 13.9% ± 5.95, respectively.

Also, the results indicated that the Kuwaiti fencers had an average maximum oxygen consumption of 49.6 ± 4.76 ml/kg/min.

The plasma lipid and lipoprotein concentration of Kuwaiti fencing players showed that they were in normal range and there were no significant differences in all values in comparison with international norms.

Blood lipids analysis did not indicate any abnormalities that present an immediate danger to the subjects' health or their physical fitness and performance. Glucose and triglycerides readings were 4.914 ± .33 mmol/L and 1.127 ± .53 mmol/L which are within the normal range for glucose and triglycerides in the blood 3.9-6.38 mmol/L and 0.40-2.50 mmol/L, respectively. Also, total cholesterol, HDL cholesterol and LDL cholesterol were in normal range of 3.87 ± .16 mmol/L, 1.057 ± .23 mmol/L and 2.32 ± .55 mmol/L, respectively. Serum hemoglobin was 15.128 ± .61 mmol/L which is in the normal range 14.0 - 17.5 mmol/L.

For the current study's subject with mean age of 21.5 years, weight of 71.1 kg, height of 175.2 cm and a moderate level of activity, the caloric estimation using Harris-Benedict formula is approximately 2655 calories per day. The subjects showed consumption of diet high in calories with the mean of 3459.2 ± 916.9 a day.

Macronutrients consumed by the Kuwaiti fencing team were compared to RDA values during the 3-day dietary record illustrated in Table 3.

**Table 3:** The energy expenditure and macronutrients intake of Kuwaiti fencers

Macronutrients	Fencing Players (mean ± SD)	Normal Range (RDA)	*P *value
Energy (Kcal)	3459.2* ± 916.9	2655 (calorie/d)	0.005

Total Carbohydrates (g/d)	393.4* ± 111.9	300 (g/d)	0.005

Total Fat (g/d)	145.4* ± 58.3	80 (g/d)	0.01

Saturated Fat (g/d)	48.8* ± 14.7	28 (g/d)	0.02

Monounsaturated Fat (g/d)	52.9* ± 16.3	34 (g/d)	0.006

Polyunsaturated Fat (g/d)	43.8* ± 18.3	17 (g/d)	0.000

Total Protein (g/d)	144.2* ± 42.3	58 (g/d)	0.000

Fiber (g/d)	14.85* ± 3.97	38 (g/d)	0.000

Cholesterol (mg/d)	467.8* ± 180.0	300 (mg/d)	0.004

* *p *< 0.05 significantly different from RDA values.

RDA = recommended dietary allowance. Established by the Food and Nutrition Board of the Institute of Medicine, the RDA is the average daily dietary intake level of a nutrient sufficient to meet the requirements of nearly all healthy individuals in a specific life stage and gender group.

The FDA estimates that the average daily intake of trans fat in the U.S. population is about 5.8 grams or 2.6 percent of calories per day for individuals 20 years of age and older.

The calories calculators based on Harris Benedict Equation and Dietary Reference Intakes, Institute of Medicine (IOM), 2005. Adapted by Mayo Foundation for Medical Education and Research.

Total carbohydrates consumed averaged 393.4 ± 111.9 g/d in comparison with normal value of 300 g/d.

The mean consumption of total fat and saturated fat by Kuwaiti fencers were 145.4 ± 58.3 g/d and 48.8 ± 14.7 g/d which surpasses the recommended daily allowances set by RDA at 80 and 28 g/d, respectively. However, they consumed more monounsaturated fat 52.9 ± 16.3 g/d and polyunsaturated fat 43.8 ± 18.3 g/d.

The subjects attained higher levels of cholesterol (467.8 ± 180.0 mg/d) than the normal requirement of 300 mg/d advised by RDA.

The results of the present study also showed that the recommended dietary protein allowances 58 g/d were also exceeded. The fencers consumed high amount of protein 144.2 ± 42.3 g/d.

The low quantity of fiber consumed by the fencers 14.85 ± 3.97 g/d in comparison to daily recommended 30 g/d by the American Dietetic Association.

**Table 4:** The Micronutrients intake of fencing players (N = 15)

Micronutrient	Fencing Players (mean ± SD)	Normal Range (RDA)	P value
Vitamin C (mg)	153.13* ± 64.3	90 mg/d	.041

Iron(mg)	20.45* ± 5.82	8 mg/d	.000

Calcium (mg)	974.8 ± 334.9	1000 mg/d	.783

Sodium(mg)	5306.6* ± 1033.9	2300 mg/d	.000

Potassium(mg)	4146.14 ± 1333.2	4700 mg/d	.144

Phosphorus (mg)	2049.71* ± 627.6	800 mg/d	.000

Caffeine (mg)	69.91* ± 55.6	25 mg/d	.01

*: *p *< 0.05 significantly different from RDA values.

There was a statistically significant difference in the values for all micronutrients consumed by the Kuwaiti fencing team and the RDA except for calcium and potassium. The subjects Vitamin -C- (ascorbic acid), consumption of 153.13 ± 64.3 mg/d exceeded the recommended daily allowances set by RDA of 90 mg/d.

There were no significant differences in consumption of calcium 974.8 ± 334.9 mg/d and the dietary recommendation quantity allowed by RDA 1000 mg/d.

The positive outcomes from the subjects diet is the adequate amount of iron consumed 20.45 ± 5.82 mg/d in comparison with recommended dietary allowance 8 mg/d. In addition, the Kuwaiti fencers have a normal amount of hemoglobin 15.128 ± .61 mmol/L in their blood. This is a result of higher consumption of iron.

The high quantity of sodium consumed by fencers (5306.6 ± 1033.9) exceeds the recommended by RDA (2300 mg/d). There was also higher phosphorus consumption 2049.71 ± 627.6 in comparison with the average daily intake 800 mg/d. There is also an increase in caffeine consumption of 69.91 ± 55.6 mg a day in comparison with RDA recommendation of no more than 25 mg/d.

There was significant difference in all macronutrients consumed by Kuwaiti fencers.

The results of table 5 show that Kuwaiti fencers consumed less carbohydrate 47.8% ± 1.7 of total calories a day and had more saturated fat 16.5% ± .84 and more total protein 16.6% ± .80 than recommended percentages.

**Table 5:** The percentages of total carbohydrates, lipids (saturated fat, monounsaturated fat and polyunsaturated fat) and protein from Kuwaiti fencers' dietary intake

Variables	Percentages (%) ± SD	Normal Range †	P value
Total Carbohydrates	47.8%* ± 1.70	55 - 65%	.000

Total Fat	35.6%* ± 1.66	25 - 35%	.000

Saturated Fat	16.5%* ± .84	7-10%	.000

Monounsaturated Fat	11.1%* ± .46	5-10%	.000

Polyunsaturated Fat	8.0%* ± .64	5-7%	.000

Total Protein	16.6%* ± .80	10 - 15%	.000

*: *p *< 0.05 significantly different from RDA values.

**† **American College of Sports Medicine - American Dietetic Association and Dietitians of Canada American Heart Association recommendation

In addition, they also consumed more monounsaturated fat 11.1% ± .46 and polyunsaturated fat 8.0% ± .64 which is considered being a healthy fat. Polyunsaturated and monounsaturated fat intake at levels up to 5-7% and 5-10% respectively, of total calorie intake per day is recommended by most nutrition experts.

The percent of total fat consumed from all calories per day was 35.6% ± 1.66 which in the normal range recommended by RDA of 25 - 35% of total calories a day.

Consumption of total protein percentage increased to 16.6% ± .80 percent from the normal range of 10 - 15% recommended by RDA for athletes such as fencers.

The results of table 6 show that the most desirable meal is lunch followed by dinner 53.9% ± 1.7 and 35.3% ± 2.1, respectively. Only 3.4% ± 1.5 of all subjects had snack throughout the day. Only 7.4% of players ate breakfast.

**Table 6:** The percentages of fencers eating breakfast, lunch, dinner and snacks

Variables	Percentages (%) ± SD
Breakfast	7.4% ± 1.9

Lunch	53.9% ± 1.7

Dinner	35.3% ± 2.1

Snacks	3.4% ± 1.5

## Discussion

Body composition was estimated by two methods, first, applying the BMI formula where the mean for Kuwaiti fencers was 23.5 which is in the normal weight range according to the health chart represented by the World Health Organization (WHO). However, the average BMI for the Kuwaiti male (over 15 years) according to WHO is 27.5, a very high indication of being overweight [26]. The health chart is represented by the following health categories: Underweight, BMI = < 18.5 Normal weight, BMI = 18.5-24.9 Overweight, BMI = 25-29.9 Obesity, BMI = 30 or greater [39]. The lower score of Kuwaiti fencers (23.5) maybe due to their daily athletic training.

The second method, using the BOD POD device illustrated Kuwaiti fencers having an average of 13.9% body fat which is over the normal range.

According to American College of Sports Medicine (ACSM's Guidelines for Exercise Testing and Prescription), the ideal percentage of body fat for a non-athlete is around 15-18% for men and for athletes (depending on the type of sport) it is less than 10%. For example, a bodybuilder's body fat levels are between 3-5% and for male soccer players, body fat percentages are between 7-12% [40].

The average percent body fat for national-class Polish fencers is 12.2% according to an earlier study [13]. Another study suggested that the fencers body composition and somatotype differ from the normal untrained individual [41]. A typical fencer should have on average of 8-12% body fat where the recommended value for healthy individuals is 15-18% according to ACSM [40]. The ideal body fat percentage for the general male population up to 30 years of age ranges between 9-15%. The American Council of Exercise suggested an average percentage body fat for athletes is 6-13%. However, the Kuwaiti fencers have an average of 13.9% body fat which is slightly over the recommended range.

Nutrition plays a key role in optimizing physical performance and recovery from strenuous exercise (American College of Sports Medicine, American Dietetic Association, and Dietitians of Canada, 2009 [1].

It is well documented that a diet rich in cholesterol, saturated fats and low in fiber consumption may lead to heart attack and cardiovascular complications. The diet consumed by Kuwaiti fencers consumed (high in cholesterol 467.8 mg/d, high in saturated fats 16.5% and low in fiber 14.8 g/d) could lead to future health problems. Although, the BMI and % body fat was in the normal range, the fencers should pay greater attention to their diet, especially in the regards to the intake of refined carbohydrates and saturated fat.

The Maximum Oxygen Consumption (*V*O_2 max_) (ml.kg^-1^.min^-1^) results for Kuwaiti fencers varied greatly with ranges between 43.20 - 60.60 ml.kg^-1^.min^-1 ^with an average of 49.6 ml.kg^-1^.min^-1^. These values were similar to those of non-athletes which are between 43-52 ml/kg/min. However, average *V*O_2 max _values for Kuwaiti fencers was less than the British average (54.8 ml.kg^-1^.min^-1^), Swedish average (67 ml.kg^-1^.min^-1^), and the average for the National Collegiate Athletic Association Division I fencers in USA (52.2 ml.kg^-1^.min^-1^) [42-44].

In addition, the average *V*O_2 max _for soccer players and gymnasts are 54-64 and 52-58 ml.kg^-1^.min^-1^, respectively [45]. Moreover, elite endurance athletes often average 70 ml/kg/min. One of the highest recorded *V*O_2 max _results (90 ml.kg^-1^.min^-1^) was that of a cross country skier [46]. The Kuwaiti fencers had an average of 49.6 ml.kg^-1^.min^-1^which is less than the average in most athletes particularly with fencers. This is may be an indication of lack of cardiovascular (aerobic) endurance training.

The results on plasma lipids showed no abnormalities in blood lipid profile. It is well documented that aerobic exercise training will improve the blood lipid profile [47,27,49,28]. This could be an indication that the players are engaged in a well designed training program.

Energy requirements and energy expenditure should be considered when designing a training program. A well-designed training program should depend on a balance between diet and energy intake [1]. Athletes who consume a balanced diet that meet energy needs can enhance physiological training adaptations. Moreover, maintaining an energy deficient diet during training may lead to loss of muscle mass and strength, increased susceptibility to illness, and may lead to overtraining.

Fencers should consume enough calories to supply the energy demand from exercise and daily body functions in order to avoid an energy deficit. However, the fencers in the present study had high caloric intake which should be monitored by coaches in order to avoid weight gain, obesity and possible nutrition related diseases.

Recent studies suggest that diet records are more valid measures of nutrient intake than are food-frequency questionnaires [50,51]. Therefore, a three-day diet record was used to estimate mean daily dietary energy, macronutrients, micronutrients intakes and total energy (calories) requirements.

Determination of food intake and analysis showed that the average Kuwaiti fencer should increase total carbohydrate consumption to meet the energy demand of training and competitions. It is important to increase and maintain high level of glycogen in the liver and skeletal muscles. Carbohydrates are important to maintain blood-glucose levels during exercise and avoid muscle glycogen depletion [52-54].

In order to increase fat loss by fencers, it is important to follow a healthy and balanced diet, which includes a wide selection of nutritious foods containing vitamins and essential minerals. The mean intake of saturated fat by Kuwaiti fencers was greater than 10% of the subject's ideal caloric level.

The high intake of total protein 144.2 ± 42.3 g/day should be reduced due to the fact that the protein selected by fencers contained a very rich saturated fat content. It should be noted that a typical Middle Eastern diet incorporates a high red meat and poultry consumption, and uses a deep fried style of cooking. This may explain the high levels of iron found in the fencers blood analysis. Lack of iron may cause anemia where hemoglobin in the blood contains a lower than normal amount of red blood cells that helps transport oxygen throughout the body. This would lead to symptoms such as shortness of breath, heart palpitations and fatigue [55].

Furthermore, when subjects were asked regarding the use of amino acid supplementation, all of them denied intake. Amino acid supplementation is not recommended for the fencers due to their high protein diet intake.

These preliminary findings in lipid-lipoprotein profiles, in conjunction with the findings of unbalanced diet consumption among fencing players, demonstrate the need for further research in this group of athletes. The results of several studies confirmed that saturated fatty acids leads to early development of CHD whereas monounsaturated and polyunsaturated fatty acids, significantly prevents the possibility of CHD [56-61]. The intake of monounsaturated fats and polyunsaturated fats were higher than the recommended values indicating appropriate choice of food yet, the diet consumption of the fencers is still high in total fat content when compared to the RDA values.

Although the blood lipids profile test revealed Kuwaiti fencers have normal blood lipids, the dietary intake analysis showed an unbalanced macronutrients and micronutrients consumption. A dietary intervention for Kuwaiti fencers by qualified and registered dietitians is needed to focus on healthy food choices and reduction of saturated fats.

Reduced fiber intakes have many health complications. The subjects in the present study have very low intake of fiber in comparison with the value recommended by all diet agencies. The low fiber intake could cause certain types of cancer and is associated with constipation, risk of heart disease and other digestive problems [62,63].

The players consumed both calcium (Ca) and potassium (K) that were marginal in comparison with recommended values, therefore, the mineral content of the foods consumed was adequate for the athlete. However, it is important to avoid any deficiencies in Ca and K. Calcium, builds bones and prevents osteoporosis. Potassium, helps muscles and nerves function properly, maintains the proper electrolyte balance, acid-base balance and lowers the risk of hypertension [1].

The high quantity of sodium consumed by fencers (5306.6 ± 1033.9) exceeds the recommended by RDA (2300 mg/d). This is mostly due to the nature of the Kuwaiti diet and high percentage of fast food consumption. The current recommendation is to consume less than 2,400 milligrams (mg) of sodium a day. This is about one teaspoon of table salt per day. It includes all salt and sodium consumed, including sodium used in cooking and at the table.

Although caffeine increases athletic performance and concentration it has adverse effects including possible anxiety, dependency, and withdrawal from the central nervous system [64-66]. Most of the caffeine consumed by the subjects is due to the social life style of Kuwaitis where tea and strong Arabian coffee is consumed as a tradition.

It is well documented that eating breakfast has many benefits [67,68]. Skipping breakfast may lead to weight gain, fatigue and other health complications [69-72]. Due to the fact that most jobs and school day ends by 12 noon, the lunch meal is largest and most desirable (53.9%). In the present study only 7.4% ± 1.9 of fencing players consumed breakfast. It is important to advise the players to eat healthy and balanced breakfast.

## Conclusion

The most significant findings of the present study is that the Kuwaiti national-class fencers had a normal blood profile, an average body fat composition, consumed an unbalanced diet and recorded a less than average VO_2 max _value in comparison to the other fencers.

The diet of Kuwaiti fencers showed an inadequate nutritional profile when compared with recommendations for healthy people by RDA. These athletes need to be educated about consuming an adequate and healthy diet to meet the nutritional needs of their activity and to avoid health problems.

The data suggest that the Kuwaiti fencers require intensive nutritional education about healthy dietary practices and proper selection of nutrients as well as behavioral modification that encourages eating breakfast daily.

The results of the present study may be used as the basis for further research such as the study of the physical fitness profiles of the Kuwaiti national-class fencers and the effect of improved dietary practices on their athletic performance.

## Competing interests

The authors declare that they have no competing interests.

## Authors' contributions

KG, the first author designed and wrote the introduction and the conclusion. SH, participated in the design of the study and performed the statistical analysis. Both authors read and approved the final manuscript.
